# The Efficacy of Therapeutic DNA Vaccines Expressing the Human Papillomavirus E6 and E7 Oncoproteins for Treatment of Cervical Cancer: Systematic Review

**DOI:** 10.3390/vaccines10010053

**Published:** 2021-12-31

**Authors:** Ayazhan Akhatova, Chee Kai Chan, Azliyati Azizan, Gulzhanat Aimagambetova

**Affiliations:** 1 School of Medicine, Nazarbayev University, Kabanbay Batyr 53, Nur-Sultan 010000, Kazakhstan; ayazhan.akhatova@nu.edu.kz; 2 Department of Biomedical Sciences, School of Medicine, Nazarbayev University, Kabanbay Batyr 53, Nur-Sultan 010000, Kazakhstan; cchan@kean.edu (C.K.C.); aazizan@touro.edu (A.A.); 3 Department of Biology, College of Science and Technology, Wenzhou-Kean University, Wenzhou 325000, China; 4 Department of Basic Sciences, College of Osteopathic Medicine, Touro University Nevada, Henderson, NV 89014, USA

**Keywords:** cervical cancer, cervical intraepithelial neoplasia, HPV, E6 oncoprotein, E7 oncoprotein, therapeutic vaccine, DNA vaccine, DNA therapeutic vaccine

## Abstract

Cervical cancer is recognized as a serious public health problem since it remains one of the most common cancers with a high mortality rate among women despite existing preventative, screening, and treatment approaches. Since Human Papillomavirus (HPV) was recognized as the causative agent, the preventative HPV vaccines have made great progress over the last few years. However, people already infected with the virus require an effective treatment that would ensure long-term survival and a cure. Currently, clinical trials investigating HPV therapeutic vaccines show a promising vaccine-induced T-cell mediated immune response, resulting in cervical lesion regression and viral eradication. Among existing vaccine types (live vector, protein-based, nucleic acid-based, etc.), deoxyribonucleic acid (DNA) therapeutic vaccines are the focus of the study, since they are safe, cost-efficient, thermostable, easily produced in high purity and distributed. The aim of this study is to assess and compare existing DNA therapeutic vaccines in phase I and II trials, expressing HPV E6 and E7 oncoproteins for the prospective treatment of cervical cancer based on clinical efficacy, immunogenicity, viral clearance, and side effects. Five different DNA therapeutic vaccines (GX-188E, VGX-3100, pNGVL4a-CRT/E7(detox), pNGVL4a-Sig/E7(detox)/HSP70, MEDI0457) were well-tolerated and clinically effective. Clinical implementation of DNA therapeutic vaccines into treatment regimen as a sole approach or in combination with conservative treatment holds great potential for effective cancer treatment.

## 1. Introduction

Cervical cancer is a largely preventable cancer of the cervix, which is the narrow part of the lower uterus that connects to the vagina. According to the World Health Organization (WHO) statistics, cervical cancer became the fourth most frequent cancer in women in 2018, with 570,000 cases, which represent 6.6% of all female cancers worldwide [1]. Similarly, cervical cancer is the 2nd most common type of cancer among females, and the 4th most common cause of cancer-related deaths (8.5%) among women in Kazakhstan [1].

Previous studies have established the strong causative association between persistent infection with certain high-risk Human Papillomavirus (HPV) types and the development of cervical cancer [2]. HPV is a small, non-enveloped deoxyribonucleic acid (DNA) tumor virus, which primarily affects human vaginal and oral mucosa [2]. There are more than 100 HPV subtypes, which differ by less than 3% of their genome [3]. The most prevalent oncogenic subtypes in both symptomatic (50–70%) and asymptomatic (20–30%) women diagnosed with invasive cervical cancer are HPV-16 and HPV-18 [3].

About 90% of deaths from cervical cancer occurred in low- and middle-income countries largely due to the lack of proper prevention, early diagnosis, and effective screening [1]. In 2018, WHO started a new campaign to decrease the incidence rate, with the aim to eventually eradicate cervical cancer [4]. The campaign included three key steps, which are vaccination of 90% of girls by 15 years of age, screening provision of 70% of women by 35 years of age and again by 45, and treatment of 90% of women with diagnosed cervical neoplasia [5]. Ideally, if all countries accomplished the requirements of the campaign by 2030, it is expected to decrease the incidence of new cases by 40% and 5 million related deaths by 2050 [1]. However, it is now estimated that the annual number of new cases of cervical cancer would be increasing to 700,000, and the number of deaths would reach 400,000 by 2030 [4]. Such an increase is explained by the uneven provision of screening and vaccination among countries, since these actions have occurred mostly in high-income settings [4]. In high-income countries, screening programs cover 60% of the female population, while, in lower-middle-income countries, the figure is only 20% [4]. Although preventative measures are expected to be effective in the elimination of cervical cancer in the long term, the situation now requires a short-term solution for those already in need of better treatment and care.

Nowadays, early-stage cervical intraepithelial lesions (CIN) are treated by means of surgical resection of cancerous tissue, which include conization, loop electrical excision procedure (LEEP), and radical hysterectomy [6]. These already traumatizing procedures can be coupled with radiotherapy or chemotherapy for the purpose of treatment enhancement and prevention of relapse [6]. Since radiotherapy and cytotoxic chemotherapy target not only cancerous tissue but surrounding tissues as well, patients often suffer constitutional side effects such as fatigue, loss of appetite, nausea, hair loss, or adverse events (AEs) that negatively impact patient’s quality of life like anemia, neutropenia, thrombocytopenia, neuropathy, nephro-/hepatotoxicity, premature menopause, and infertility [6]. Therefore, it is necessary to provide less toxic and traumatic treatment options, especially for patients with comorbidities. After surgical excision, quadrivalent HPV vaccination could be used for CIN2+ cervical lesions to reduce the risk of recurrent disease [7].

Therapeutic vaccines such as TheraCys, PROVENGE, and IMLYGIC used for the treatment of urothelial carcinoma in situ, prostate cancer, and advanced melanoma respectively with promising results [8]. These cancer vaccines showed greater median overall survival compared to the conservative chemotherapy approaches, resulting in the United States Food and Drug Administration (FDA) approval [8]. The development of effective therapeutic vaccines for precancerous cervical lesions and cervical cancer treatment and their implementation into clinical practice would be a huge improvement in gynecologic oncology.

Currently, HPV therapeutic vaccines under investigation include live vector vaccines (bacterial and viral vectors), subunit vaccines (peptides and protein-based vaccines), plant peptide/protein-based vaccines, nucleic acid vaccines (DNA and ribonucleic acid (RNA) replicon-based vaccines), and cell-based vaccines (dendritic cell-based vaccines and adoptive cell transfer) [2]. Among these subtypes, we are particularly interested in DNA therapeutic vaccines, since they are safe, cost-efficient, thermostable, easily produced in high purity, and distributed [2]. Unlike live vector vaccines, DNA therapeutic vaccines do not evoke neutralizing antibody production, thus allowing for repeated vaccination [9]. T cell-mediated immune response is achieved by targeting HPV E6 and E7 proteins, as they are solely responsible for the malignant transformation of cervical tissue [9]. Moreover, in a study by Daayana et al., strong adaptive immune responses to E6 and E7 were reported, and it was shown to be greater than previously reported immune responses to therapeutic HPV vaccines [2,10]. Thus, the immunogenicity of DNA vaccines expressing the HPV E6 and E7 oncoproteins needs to be further investigated. Currently, the majority of DNA therapeutic vaccines are undergoing clinical trials to evaluate their safety and stability. Therefore, the aim of this article is to assess and compare existing DNA therapeutic vaccines, which are evaluated in phase I and II trials, expressing HPV E6 and E7 oncoproteins for the prospective treatment of cervical cancer based on clinical efficacy, immunogenicity, viral clearance, and side effects.

## 2. Materials and Methods

This systematic review was conducted according to the Preferred Reporting Items for Systematic Reviews (PRISMA) statement [11]. The study was registered in the PROSPERO database and confirmed with a registration code of CRD42021251476.

### Systematic Literature Search and Eligibility Criteria

Articles were manually searched using databases as PubMed/MEDLINE, Google Scholar, and clinicaltrials.gov published in English from the year 2010.

The search was performed using the following keywords: “cervical cancer”, “cervical intraepithelial neoplasia”, “HPV”, “HPV-positive”, “E6 and E7 oncoproteins”, “therapeutic vaccine”, “DNA vaccine”, and “DNA therapeutic vaccine”. We used the medical subject heading (MeSH) term “Uterine Cervical Neoplasms” (MeSH Unique ID D002583) as major topic and “Vaccines” (MeSH Unique ID D014612), “E6 protein, HPV type 18” (MeSH Unique ID C052603) and “E7 protein, HPV type 16” (MeSH Unique ID C059731).

The search was narrowed by using “Cervical cancer OR Cervical Intraepithelial Lesion AND DNA therapeutic vaccines”, “DNA therapeutic vaccines AND E6 OR/AND E7 oncoproteins”. The selected studies were independently reviewed for inclusion eligibility by two reviewers (Akhatova and Aimagambetova) using standardized data collection forms. The following data were collected from the studies: the author, year of publication, number of study participants, vaccine administration strategies, and the main outcomes (clinical efficacy, viral clearance, immunogenicity, adverse events). Any discrepancy in the assessment of articles was resolved by discussion and consensus, as well as input from the third and fourth reviewers (Chan and Azizan).

The articles were selected to meet the following eligibility requirements to be included in the study: (1) research article, (2) human subject research, and (3) the study of DNA therapeutic vaccines targeting HPV E6 and E7 oncoproteins. The presence of the following did not allow for the study to be included: (1) reviews and case reports, (2) irrelevance to cervical cancer or CIN, (3) mouse model studies, (4) articles on preventative HPV vaccines, and (5) the use of the inappropriate methodology. Abstracts lacking full information about predefined criteria were excluded without further review.

The types of studies included were phase I and phase II clinical trials that were initiated and completed between 2003 and 2017, studying the clinical efficacy of DNA therapeutic vaccines expressing HPV E6 and E7 oncoproteins for the treatment of cervical intraepithelial lesions of grades 2 and 3 both newly diagnosed and recurrent malignancies. The treatment of the lesion may or may not be followed by conization or loop electrosurgical excision procedure. The study population was female patients aged 18 or older with histopathologically diagnosed CIN of grades 2 and 3, known to be caused by HPV 16 and/or HPV 18 based on polymerase chain reaction (PCR) amplification results. The main outcomes of the review were clinical efficacy based on the lesion regression, viral load reduction, immunogenicity, in particular, HPV E6 and E7 specific CD8+ T cell response, and AEs after vaccination.

## 3. Results

### 3.1. Study Identification and Selection

**Figure 1:**
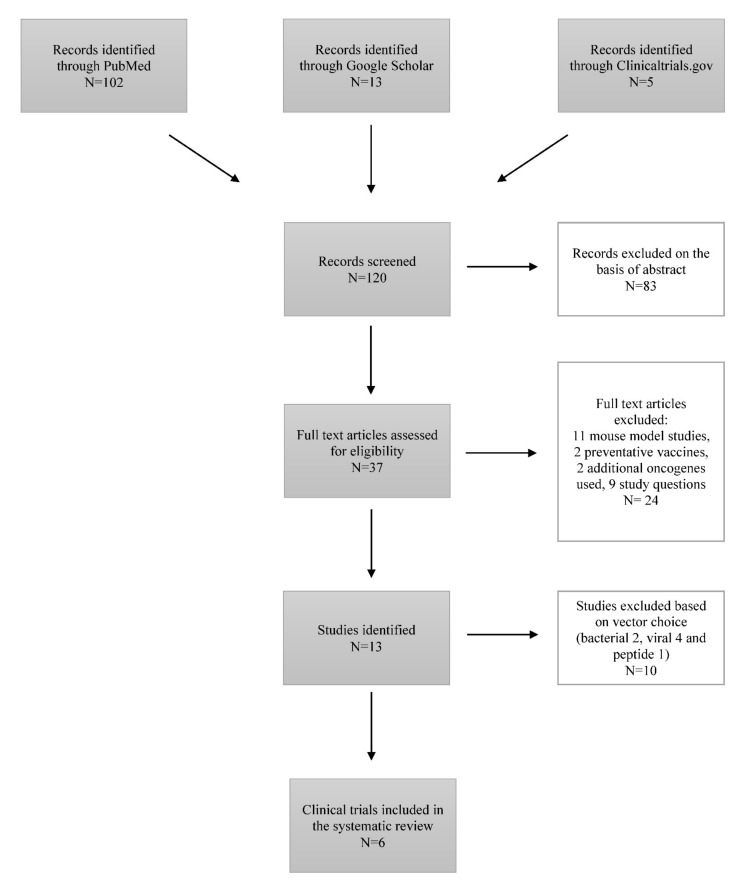
Flow-chart diagram of study selection.

During this study, 120 articles were identified through PubMed/MEDLINE and Google Scholar searching platforms (Figure 1).

Eighty-three articles were excluded based on the abstract, representing literature reviews. From the remaining 37 articles, 24 articles were excluded at this stage: 11 studies were mouse model-based, 9 studies were not addressing the study question, 2 studies were regarding the preventative vaccines, and 2 studies included additional oncogenes in the development of therapeutic vaccines. We included 6 studies performed between 2003 and 2017 in our systematic review after the exclusion of 7 articles studying the effects of therapeutic vaccines with either viral, bacterial, or peptide vectors [12,13,14,15,16,17]. These six studies represented the work completed in the United States of America, Korea, Estonia, South Africa, India, Canada, Australia, and Georgia [12,13,14,15,16,17] (Table 1).

Therapeutic vaccines were evaluated based on clinical efficacy (histopathological regression of the lesion to CIN < 1), viral clearance, immunogenicity, and adverse events after the vaccination. Subjects, female patients aged 18 or older with histopathologically diagnosed cervical intraepithelial neoplasia of grades 2 and 3, known to be caused by HPV 16 and/or HPV 18, received DNA therapeutic vaccines in different dose formulations and were evaluated generally within 20 weeks (36 weeks in extended trial groups) for the effects of vaccines mentioned above. Study populations ranged from 10 patients to 167, median age mostly being 21–30, except for Hasan et al. (2020) study, where the median age of participants was 51.50 years old due to more advanced stages of cancer [17].

### 3.2. Outcomes

#### 3.2.1. Clinical Efficacy

Clinical efficacy was evaluated according to the histopathological regression to CIN ≤1, which is less than one-third of the thickness of the cervical epithelium, on a colposcopy-guided biopsy 15, 20, or 36 weeks after the first injection. All six studies [12,13,14,15,16,17] report tumor size decrease to some extent (Table 2).

GX-188 E in phase I trial by Kim et al. [13] showed a 78% success rate of complete response both histologically and virologically. The same vaccine in the phase II trial by Choi et al. (2019) resulted in histopathological regression to CIN < 1 in 52% of patients 20 weeks and 67% of patients 36 weeks after the first dose [12]. MEDI0457 by Hasan et al. showed 87.5% of complete response to the vaccine and 1 patient had a partial response to the treatment [17]. pNGVL4a-CRT/E7(detox) [15] and pNGVL4a-Sig/E7(detox)/HSP70 [16] vaccines both showed a similar response rate of around 30%.

#### 3.2.2. Viral Load Clearance

Viral load was measured by means of PCR amplification to assess the clearance of HPV DNA from the cervical biopsy after vaccination. Choi et al. established that HPV clearance was associated with the histopathologic regression as 77% of regressors had no trace of HPV DNA, while only 12% of non-regressors had no viral load in the tissue biopsy [12]. Kim et al. results show that GX-188E takes time to clear off the virus [13]. MEDI0457 [17] and VGX-3100 [14] report the association between viral clearance and tumor size reduction, whilst pNGVL4a-CRT/E7(detox) [15] did not result in any difference between pre- and post-treatment viral load.

#### 3.2.3. Immunogenicity

Immunogenicity is one of the key features of the therapeutic vaccines as it represents the potential of the vaccine to induce virus-specific T cell response, in particular HPV E6 and E7 specific CD8+ T cell immune response. IFN-γ response was measured by means of *ex vivo* ELISpot assay with cryopreserved and thawed peripheral blood mononuclear cells (PBMCs) at pre- and post-treatment stages. The vaccine response is considered positive when the increase in T-cell frequency was at least three times greater compared to the study entry measurement. GX-188E both in phase I [13] and phase II [12] studies showed a significant increase in IFN-γ response, which was correlated with the histopathologic regression and viral clearance. Moreover, an E6 specific response was more pronounced than E7 specific [12,13]. VGX-3100 induced 9.5 times greater IFN-γ response in the treatment group compared to the placebo, which lasted as long as 24 weeks post-vaccination [14]. On the contrary, MEDI0457 induced a greater response to E7, particularly in newly diagnosed cohort 1 that persisted up to 48 weeks [17]. In cohort 1, 4 of 7 patients exhibited IFNγ-producing spots exceeding 100 SFU/10^6^ PBMC, whereas no patients produced similar responses in cohort 2 [17]. pNGVL4a-CRT/E7(detox) and pNGVL4a Sig/E7(detox)/HSP70 showed minimal dose-dependent immune response, which was remarkable from the unvaccinated group [15,16].

#### 3.2.4. Toxicity/Adverse Events

Overall, all vaccines were well-tolerated without vaccine-related serious adverse events. The most common adverse events were injection site pain and erythema, as well as constitutional symptoms (malaise, myalgia, and headache) [12,13,14,15,16,17]. No serious adverse events (Grade 3/4) related to the vaccination were reported. No dose-limiting toxicities were observed.

## 4. Discussion

This systematic review summarizes the findings of phase I and phase II clinical trials investigating the treatment of patients with histopathologically diagnosed CIN associated with HPV 16 or/and HPV 18 with DNA therapeutic vaccines. Six studies have demonstrated immunologic response in the form of lesion size regression, viral clearance, and increased T cell response of five different DNA vaccines–GX-188E (phase I and phase II), VGX-3100, pNGVL4a-CRT/E7 (detox), pNGVL4a-Sig/E7 (detox)/HSP70, MEDI0457. Vaccines were plasmid DNA encoding for either non-oncogenic E6/E7 or both, and chaperonin proteins such as HSP 70 and Calreticulin for the enhancement of the uptake by antigen-presenting cells, and MHC class I processing and presentation. MEDI0457 [17] had the same plasmid formulation as VGX-3100 [14] combined with plasmid encoding IL-12. All vaccines were well tolerated by patients, leading to only grade 1 or less systemic and local side effects.

Previous reviews have studied various existing therapeutic vaccines including live vectors, plant-based, protein, whole cell, and combinatorial vaccines [18]. This is the first systematic review of DNA therapeutic vaccines against cervical cancer expressing HPV16 and HPV18 E6 and E7 oncogenes. The feasibility of production, storage, and transportation, cost-effectiveness, the capability of multiple immunizations, and targeting different co-stimulatory genes provided the rationale for the study of DNA therapeutic vaccines [18]. However, comparatively weak immunogenicity and the risk of integration into the host genome are the main concerns, which could be addressed by modification of E6 and E7 to abolish its transformative capacity [18]. There are approaches of boosting the potency of DNA vaccines, such as increasing the number of antigen-expressing dendritic cells (DCs) by using a gene gun delivery method, enhancing antigen processing and presentation in dendritic cells via codon optimization, and improving the DCs and T-cell interaction [18]. These strategies were used in our selected studies, which led to increased antigen-specific, activated CD8+ T cell response in all of them. Patients with CIN2/3 were more likely to induce E6 and E7 specific CD8+ immune response, according to the IFNgamma ELISPOT results, compared to the invasive cervical cancer [17]. According to Hasan et al., diminished immune response in more advanced disease stages is associated with immune exhaustion, the effect of chemoradiation and selection of patients with diminished immunity against HPV [17]. The strongest evidence of the immunogenicity of DNA therapeutic vaccine VGX-3100 was observed by the increased intensity of CD8+ infiltrates in histopathologically regressed patients compared to the placebo group with regressed lesions [14].

DNA therapeutic vaccines were also assessed based on their clinical efficacy, i.e., the ability to induce cervical lesion regression. The regression to ≤CIN1 among study participants was observed in all studies with significantly varying degrees. The study of VGX-3100 vaccine with both treatment and placebo groups showed a response rate of 49.5% vs. 30.6%, respectively [14]. Meanwhile, GX-188E vaccine has resulted in histopathological regression in 67% of patients in both phase I study and phase II studies [12,13]. Choi et al. [12] have observed an enhanced response to GX-188E over time up to 83% among those with cervical lesions <50%, probably due to the enhanced memory T cell-driven therapeutic effect. The difference in clinical benefit between VGX-3100 and GX-188E could be explained with the recruitment of CIN3 HPV-positive patients only, the lack of placebo group, and the small number of participants in the latter. pNGVL4a-CRT/E7 (detox) and pNGVL4a-Sig/E7 (detox)/HSP70 had the lowest clinical efficacy of approximately 30% response rate among all [15,16]. However, the effect of these two vaccines on the lesion regression is questionable, as this rate is similar to spontaneous remission rate over a 15-week period [15].

It was established that women, after excision of the cervical lesion, are more likely to have a relapse; therefore, viral clearance is a key factor of vaccine efficacy [18]. VGX-3100, GX-188E, and MEDI0457 effectively cleared detectable HPV DNA, which was significantly associated with histopathological regression [12,13,14,17]. In contrast, pNGVL4a-CRT/E7 (detox) has not resulted in viral load reduction [15].

Nevertheless, there are several limitations to this study. Firstly, limited data exist on the topic of DNA therapeutic vaccines, as not a single therapeutic vaccine against cervical cancer was approved. All these clinical trials were in either the phase I or phase II stage of assessing the efficacy and safety in humans. Secondly, the majority of studies enrolled a small number of participants without masking, stratification, or the control group, which poses a potential risk for bias. As vaccines investigated in this study had different structural designs, it was not feasible to make a statistical analysis of vaccine outcomes; therefore, qualitative analysis was performed overall.

## 5. Conclusions

As it was stated by WHO, a global strategy to accelerate the elimination of cervical cancer, 90–70–90 targets for prevention, screening, and treatment are the key to success. Preventative bivalent and quadrivalent HPV vaccines have undergone significant advancement in development and implementation. However, these preventative vaccines do not elicit a therapeutic effect but could be used as an adjuvant to surgical treatment. DNA therapeutic vaccines represent a potentially safe and novel approach to cervical cancer treatment. The main goal of this review was to discuss the effectiveness of existing DNA therapeutic vaccines against cervical cancer expressing HPV 16/18 oncoproteins E6 and E7. The idea of DNA therapeutic vaccines is inducing an adaptive immune response and immunologic memory via the expression of tumor antigens and activation of antigen-presenting cells.

DNA therapeutic vaccines are currently undergoing clinical trials to improve the potency of therapeutic vaccines and clinical efficacy using strategies as a modifying route of administration, adjuvant therapy, prime-boost regimen, and co-administering with other drugs for a synergistic effect. Nowadays, despite the treatment of locally advanced disease with chemoradiation, patients have a high recurrence rate and a poor 5-year survival rate, estimated at 50% and 70%, respectively. In contrast, the MEDI0457 vaccine, which contained VGX-3100 plasmid coupled with an IL-12 expression plasmid to promote T-cell function, evaluated the disease progression-free survival (PFS) at 12 months, which was estimated as 88.9% overall. These findings strengthen the hypothesis that DNA therapeutic vaccines could effectively induce *de novo* or boost existing immune responses. Moreover, studies have shown that using femtosecond laser treatment could also improve transfection efficiency administered intradermally and into the lesion *in vivo*. Thus, continuous efforts to improve the efficacy of DNA therapeutic vaccines and implementation of therapeutic vaccines into a treatment regimen as a sole approach or in combination with conservative treatment may greatly improve the current situation.

## Figures and Tables

**Table 1:** Clinical trials.

Vaccine	Trial Design	Pt.(N)	Site/ Stage	Study Type	Vaccine Type	Additional Therapies	HPV Positivity assessment	Study Duration/Location	Study Status	Clinicaltrials.Gov Identifier
**GX-188E** **[12]**	Subjects–19–50 years old women with HP diagnosed with CIN3 from an HPV type 16/18 (+), randomly assigned to treatment groups and received either 1 or 4 mg of GX-188E IM by EP in the deltoid muscle. Drug administration was performed 3 × in total during study period at visits 2, 3, and 5 (weeks 0, 4, and 12). At weeks 14 and 20 after the initial GX-188E administration, the efficacy of GX-188E was evaluated by CB and HPV DNA test. After 20 weeks of study, patients were provided with the option of entering the extension study for total of 36 weeks. *Primary outcome:* the rate of participants with HP regression to CIN ≤ 1 at V7 [10]. *Secondary outcome:* the rate of participants whose result inverted negative in HPV DNA test, the rate of HPV E6, E7-specific ELISPOT responder, cytological changes of the cervical lesions, the rate of AEs and solicited AEs, data in physical examination, vital signs, ECG, clinical lab test results related to investigational product, mean value of visual analogue scale on pain intensity, Flt-3L serum concentration.	72	CIN 3	Prospective, randomized, multicenter, open-label, phase II trial	HPV E6/E7 DNA therapeutic vaccine (Genexine, Inc.), consisting of a tissue plasminogen activator signal sequence, an FMS- like tyrosine kinase 3 ligand, and shuffled E6 and E7 genes of HPV type 16/18, as described previously.	None.	PCR identification	Trial conducted at 4 Korean sites: the Catholic University of Seoul St. Mary’s Hospital (Seoul, South Korea), the Cheil Hospital (Seoul, South Korea), the Korea University Guro Hospital (Seoul, South Korea), and the Keimyung University and Dongsan Hospital (Daegu, South Korea) for 36 weeks. *Study Start date*: July 2014. *Study Completion date:* March 2016.	Completed	NCT02139267
**GX-188E** **[13]**	Subjects–20–50 year old women with HP diagnosed HPV16/18-associated CIN3 were vaccinated in a series of three injections IM using EP device in deltoid muscle at weeks 0, 4 and 12. A standard 3+3 dose escalation scheme was followed and dose levels of 1, 2 and 4 mg (2 + 2 mg) were tested within 36 weeks of follow up. *Primary outcome:* determination ofMTD, clinical lab test results, vital signs. *Secondary outcome:* the expression levels of GX-188E in blood, immunologic reactogenicity by measuring HPV E6 and E7 specific T cell response by ELISPOT, and changes of the involved lesions and HPV infection status.	11	CIN 3	Open label, single center, dose-escalation, phase I study	A plasmid DNA encoding E6 and E7 proteins of HPV E6 or E7 genes fragmented into two parts (C-terminal and N-terminal regions) with a small overlapping sequence (encoding 16 amino acids). The fused DNA sequences including tpa, Flt3L and shuffled E6/E7 genes were inserted in high expression vector, pGX27 produced in *E. coli* DH5alpha under cGMP condition.	None.	PCR identification.	Cheil General Hospital & Women’s Healthcare Center, Seoul, Korea for total of 36 weeks. *Study start date:* November 2012. *Study Completion Date:* February 2014.	Completed.	NCT01634503
**VGX-3100** **[14]**	Subjects-18–55 years old women with HP confirmed HPV 16/18-positive CIN 2/3,randomized to receive 6 mg VGX-3100 (3 mg plasmid targeting HPV-16 E6 and E7, and 3 mg plasmid targeting HPV-18 E6 and E7) or placebo (1 mL), given IM at weeks 0, 4, and 12 weeks, followed by EP with CELLECTRA-5P. Randomization was stratified by age (<25 and >25 years) and CIN2 vs. CIN3. *Primary outcome:* number of participants with HP regression to CIN1 or normal pathology 36 weeks after first dose. *Secondary outcome:* number of participants with virologically proven clearance of HPV 16 or 18 in combination with HP regression of cervical lesions to ≤CIN1.	167	CIN 2/3	Multicentre, randomized, double-blind, placebo-controlled phase 2b trial with masked endpoint acquisition and adjudication.	Two DNA plasmids encoding optimised synthetic consensus E6 and E7 genes of HPV-16 and HPV-18, using a proprietary design strategy, SynCon (Inovio Pharmaceuticals, Plymouth Meeting, PA, USA).	At the week 36 primary endpoint visit, patients with colposcopic evidence of residual disease underwent standard therapeutic resection.	Linear Array HPV assay (Roche, Basel, Switzerland).	Trial conducted at 36 academic centres and private gynaecology practices in the USA, Estonia, South Africa, India, Canada, Australia, and Georgia. *Study Start date:* April 2011. *Study Completion date:* April 2015.	Completed.	NCT01304524
**pNGVL4a-CRT/E7(detox)** [15]	Subjects-≥19 years old women with HP confirmed HPV16 associated CIN 2/3 were enrolled and administered pNGVL4a-CRT-E7(detox) by either PMEDIM, or IL injection at study weeks 0, 4, and 8. LEEP or cold knife conization was performed at week 15. Patients were assessed for the safety and feasibility of vaccine administration, the clinical response, and the induction of an immune response to the vaccine antigen. *Primary outcome:* the feasibility and toxicity of vaccination in women with CIN2/3 caused by HPV16, evaluate the effect of vaccination on histology, comparison of immunogenicity of three different routes of administration: PMED, IM, and IL. *Secondary outcome:* changes in HPV VL, cellular immune response, humoral immune response, local tissue immune response, and correlated measures of immune response.	132	CIN2/3	Intervent., non-randomized, open label, phase I study	pNGVL4a expression vector containing coding sequences for HPV16 E7 linked to CRT. The E7 sequence in this construct has been modified at aa24 and 27, which abrogates its transforming potential. CRTis a 46 kDa calcium-binding chaperonin related to the family of HSPs.	A standard therapeutic resection of the cervical squamocolumnar junction (either a cold knife conization or a LEEP) was performed at study week 15, seven weeks after the third vaccination.	HPV16-specific TaqMan kinetic PCR amplification.	University of Alabama at Birmingham, Johns Hopkins Outpatient Center, Johns Hopkins Bayview Medical Center (US). *Study Start date:* September 2009. *Study Completion date:* July 2016.	Completed.	NCT00988559
**pNGVL4a-Sig/E7(detox)/HSP70** **[16]**	Subjects–18–50 years old women with HP confirmed HPV16 + CIN2/3 received 3 vaccinations with 1 of 3 doses of study vaccine, 0.5, 1.0, or 3.0 mg IM at weeks 0, 4, and 8, and standard therapeutic resection of the cervical SCJ at week 15. *Primary outcome:* the feasibility and toxicity of pNGVL4a-Sig/E7(detox)/HSP70 DNA vaccine in preventing the cervical cancer in HPV16+ CIN2/3, the effect of the vaccine on the histology of cervical tissue specimens from these patients. *Secondary outcome:* the changes in lesion size and HPV VL, cellular, humoral, and local tissue immune responses, correlated measures of immune response with clinical response, correlated response with those observed in the preclinical model.	16	CIN2/3	Intervent., single-site, open label, phase I dose escalation study	A closed circular DNA plasmid expressing HPV16 E7 mutated at aa 24 and 26, linked to coding for Sig and HSP70.	Patients underwent standard cone or LEEP resection of the SCJ at week 15, and had a postoperative exam at week 19.	HPV16-specific TaqMan real-time PCR method.	Sidney Kimmel Comprehensive Cancer Center at Johns Hopkins. *Study Start date:* November 2003. *Study Completion date:* January 2010.	Completed.	NCT00121173
**MEDI0457 (INO-3112)** [17]	Subjects–18–70 years old women with inoperable cervical cancer, stage IB-IVB, HPV16/18+. Patients were stratified into 2 cohorts: (1) newly diagnosed cancers; (2) persistent or recurrent cervical cancer. After chemoradiation, patients received MEDI0457 immediately followed by EP with the CELLECTRA 5P device given every 4 weeks for a total of 4 doses. *Primary outcome:* safety and tolerability of immunotherapy with MEDI0457 when delivered IM followed by EP in study patients. *Secondary outcome:* cellular and humoral immune responses to HPV 16/18 E6/E7 and treatment response as measured by clinical examination and PET/CT imaging after CRT and DNA vaccination.	10	SCC, AC, or ASCC of the cervix, stage IB-IVB	Intervent., non-randomized, open label, phase 1/2a study	Combined plasmids encoding modified, nononcogenic E6 and E7 viral oncoproteins of HPV16 and HPV18 (VGX-3100) with a plasmid encoding IL-12 (INO-9012).	CRT must have been completed within 10 weeks of initiation. Intracavitary or interstitial brachytherapy was delivered. Weekly cisplatin chemotherapy (40 mg/m^2^) was administered on day 1 of EBRT and given during weeks 1 to 5 of standard EBRT and during the parametrial boost.	ThinPrep testing for HPV PCR amplification.	University of Chicago Medical Center, University of Michigan, Columbia University Medical Center. *Study Start date*: June 2014. *Study Completion date:* September 2017.	Completed.	NCT02172911

Abbreviations: histopathological—HP; intramuscularly—IM; electroporation—EP; cervical biopsy—CB; adverse events—AEs; maximum tolerated dose—MTD; particle-mediated epidermal delivery—PMED; cervical intralesional—IL; positron emission tomography—PET; computer tomography—CT; calreticulin—CRT; heat shock proteins—HSPs; squamocolumnar junction—SCJ; viral load—VL; chemoradiotherapy—CRT; squamous cell carcinoma—SCC; adenocarcinoma—AC; adenosquamous cell carcinoma—ASCC; loop electrosurgical excision procedure—LEEP; external beam radiation therapy-EBRT.

**Table 2:** Comparison of the trials’ results.

Vaccine	Clinical Efficacy (Histopathology, Colposcopy, Tumor Size)	Viral Clearance	Immunogenicity (E6 and E7 Specific CTL Activity)	Adverse Events/Toxicity	Additional Findings	Limitations
**GX-188E,** **[12]**	HP regression to CIN < 1 in 33/64 patients (52%) at V7, and 35/52 (67%) at V8 (Visit 8, week 36). Lesions that cover <50% showed better efficacy than the ones >50% after GX-188E injection, 63% vs. 41% (V7; x2 test; P 1⁄4 0.133.	Of the patients with HP regression, 73% (24/33) exhibited HPV clearance at V7 and 77% (27/35) exhibited clearance at V8. Of the nonregressors, 16% (5/31) exhibited HPV clearance at V7 (Visit 7, week 20) and 12% (2/17) exhibited clearance at V8. HPV clearance and HP regression were significantly associated at the V7 [OR 1⁄4 13.867; 95% confidence interval (CI), 4.070—47.249; *p* < 0.001] and V8 visits (OR 1⁄4 25.313; 95% CI, 4.750–14.883; *p* < 0.001).	A higher percentage of the patients (16/25) with HP regression exhibited > 3-fold increase in IFN-γ ELISpot responses compared with the group without HP regression (x^2^ test (P 1⁄4 0.028), but 7 of 22 nonregressed patients developed more than 3-fold increase in these responses. Patients with HPV clearance (n 1⁄4 26) presented significant increases in IFN-γ ELISpot responses compared with those without clearance (n 1⁄4 21; fold changes were 28 and 10, respectively; *t* test; P 1⁄4 0.002).	GX-188E-well- tolerated. The AEs relating to the injection site-pain, erythema, induration, and swelling/edema in both groups; pain was the most common AE (occurring in 94.4% and 100.0% in the 1 and 4 mg GX-188E groups, respectively) One patient was lost to follow up due to pregnancy (1 mg GX-188E group).	HPV sequence variants: HP regression in 42% (11/26) of the CIN3 patients with HPV variants, whereas 75% (12/16) occurred in those without any of the three variants. 1 vs. 4 mg: 1 mg was found to have better efficacy at V7 and V8. (x2 test; P 1⁄4 0.006 and P 1⁄4 0.027, respectively) HLA types: HLA- A02 was associated with HP regression at V7 (20 weeks after the first injection; P 1⁄4 0.032; OR 1⁄4 2.381; 95% CI, 1.064–5.327), but not at V8 (36 weeks after the first injection; P 1⁄4 0.404; OR 1⁄4 1.490; 95% CI, 0.582–3.811.	Lack of control/placebo group. The selection bias-patients recruited into the study were diagnosed with CIN 3 only. The attrition bias-20/72 participants withdrew from the study due to various reasons. The confirmation bias—in the discussion part, authors concluded that immunologic response and HP regression had weak association. However, earlier in the results they mentioned an association between HP regression and systemic immune response.
**GX-188E** **[13]**	At 8 weeks post last vaccination (VF1), 6/9 patients were free of lesions—2 patients from each cohort (A01 and A03 from 1 mg cohort, A05 and A06 from 2 mg cohort, A07 and A08 from 4mg cohort). GX-188E vaccination led to the clinically and virologically meaningful complete response rate of 78% (7/9).	At week 12, 5/9 patients showed viral clearance. At week 20, 6/9 patients showed viral clearance. At week 36, 7/9 patients showed viral clearance.	All subjects exhibited a marked increase in the vaccine- induced E6- and E7-specific IFN-g ELISPOT response compared with the background level before vaccination. Vaccine-induced cellular immune responses became progressively stronger in all patients during GX-188E vaccination. The response against the E6 antigen was more vigorous than against E7 as determined by the magnitude of response (69–89% against E6 versus 11–31% against E7 at VF1). GX-188E vaccination-induced E6/E7- specific memory T-cell response can be maintained for at least 24 weeks post last vaccination. Apart from patient A04, GX-188E vaccine elicited activation of both HPV16-specific CD4 and CD8 T cells. The amount of Th1 effector cytokines, such as IFN-γ, IL-2 and tumour necrosis factor α (TNF-α) increased after vaccination in most of the patients (median 49.9−, 13− and 22.9−fold increases for IFN-γ, IL-2, and TNF-α, respectively).	AEs associated with GX-188E vaccination-chills, injection site pain, swelling and hypoaesthesia in 19/49 patients. AEs-headache, rhinitis and fatigue in 7/49 of the cases could be potentially associated with the vaccine.	6/7 responders carrying HLA-A*02 exhibited high polyfunctional CD8 T-cell responses as well as complete regression of CIN3. Among the two non-responders, patient A04 with HLA-A*26 and -A*30 did not induce HPV-specific CD8 T-cell responses at all.	Too small study population, which does not allow for generalization of the results and drawing conclusions. No stratification by age, ethnicity, monoinfection/mixed infection. Randomization or masking of the population was not introduced as well. No control group. All patients had CIN 3 –both severe dysplasia and carcinoma in situ (selection bias). The confirmation bias)-no consideration of spontaneous regression.
**VGX-3100** **[14]**	HP regression in 53/107 patients (49.5%) in treatment group, 11/36 (30.6%) in placebo group (PPD 19·0, 95% CI 1·4–36·6; *p* = 0·034). *Modified intention-to-treat analyses:* HP regression in 55/114 patients (48.2%) in treatment group, 12/40 (30%) in placebo group. (percentage point difference 18·2, 1·3–34·4; *p* = 0·034) *Post-hoc efficacy analyses:* HP regression to normal in 43/107 patients (40.2%) in treatment group, 6/36 (16.7%) of placebo recipients (PPD 23·5, 95% CI 4·4–37·0; *p* = 0·012).	Viral clearance occurred in 56/107 patients (52·3%) in treatment group, 9/35 patients (25.7%) in placebo group (percentage point difference 26·6, 95% CI 6·8–42·2; *p* = 0·006). Among those with HP regression, viral clearance was more likely among VGX-3100 recipients (about 80%) than among placebo recipients (about 50%).	In post-hoc immunological analyses, T-cell responses to HPV-16 and HPV-18 E6 and E7 peaked at week 14 for VGX-3100 recipients, with a 9.5 times greater median response than in placebo (*p* < 0·0001). VGX-3100 elicited significantly increased frequencies of antigen-specific, activated CD8+ T cells, identified by cell surface expression of CD137, that also expressed perforin compared with placebo (*p* = 0·001). VGX-3100 recipients with HP regression and viral clearance developed antibody responses to both HPV-16 and HPV-18 E7 that were significantly higher than for non-regressors, at the time of peak response (post-dose 3) but also as early as post-dose 2 and as late as week 24.	Injection site erythema—98/125: 78.4% in treatment group, 57.1% in placebo group. 4 patients discontinued due to AEs—2 injection site pain, 1 maculopapular reaction, 1 allergic reaction. No serious AEs reported.	None	Skewing of the population towards more severe disease and older age. 92.8% of the participants had genotype of HPV 16 + at the entry. The attritition bias-18 patients in treatment group and 6 patients in control group were excluded from the study due to different reasons. HPV genotyping, which was based on the cervical swabs, included the possibility of only HPV16 or/and HPV18. Therefore, mixed infection study group could be underestimated. The confirmation bias-Vaccination induced HP regression and viral clearance in about 40% of women with CIN2/3 positive for HPV-16 or HPV-18, whereas surgical excision would have eliminated the dysplastic tissue in 85–90% of women.
**pNGVL4a-CRT/E7(detox)** **[15]**	HP regression in 8/32 patients (30%). Remaining 70% of patients had persistent CIN 2/3. 1 patient had regression to CIN1. 7 patients had no residual CIN.	No differences between pre- and post-vaccination viral loads in any of the treatment cohorts.	Immune response to E7 was minimal and was not significantly different than response to E6. Intraepithelial CD8+ T cell infiltrates increased after vaccination in intralesional administration cohort (*p* = 0.0313).	Total vaccine specific AEs in 22/32 patients (69%). 55% of IM vaccination patients, 80% of PMED patients, and 73% of intralesional vaccination patients experienced AEs. Most common–constitutional and injection site grade 1 or less AEs. No grade 3 or 4 AEs. No vaccine-related serious AEs. 1 bleeding after LEEP, 3 pregnancies unrelated to vaccine.	None	This was a small phase I trial designed to primarily evaluate the feasibility and safety of pNGVL4a-CRT/E7(detox). Only HPV 16 positive CIN patients were included in the study. The majority of these patients were Caucasians. Patients were required to have a hemoglobin of 9 g/dL or greater. The selection bias-anemia is considered strong prognostic factor. The ND10 PMED has a reduced number of components to ease large-scale manufacturability, compared to previously used ND5.5. This could potentially lead to discrepancies in results due to device error.
**pNGVL4a-Sig/E7(detox)/HSP70** [16]	No HP progression was observed. 3/9 patients (33%) had complete histologic regression of disease at week 15 in the highest dose cohort.	NA	E7 specific T cell response was identified in 3/15 patients: 1 patient–response increased subsequent to vaccination at week 15 1 patient–stable response 1 patient–declined response. E6 specific T cell response: 5/15 patients. Overall, responses to E6 were not of greater absolute magnitude in regressors compared with non- regressors at either of the two time points. (*p* = 0.4228 and *p* = 0.4964, respectively). At 6 months response to E7 was detected in 5/9 patients (55.6%) in highest dose cohort.	Transient local reactogenicity was reported in 5/15 (33%). Systemic symptoms (malaise, myalgia, headache) after vaccination were also reported by 5/15 subjects. No dose-limiting toxicities were observed.	None	This was a small phase I study–15 patients only. No masking. Follow up period was 19 weeks, whereas the average follow-up period in selected studies was 36 weeks. Vaccine targets specifically HPV16 E7 oncoprotein, without HPV 18, or E6 oncoprotein. Local and systemic AEs were assessed by patients, which may result in self-reporting bias such as social desirability or recall bias.
**MEDI0457** **[17]**	All cohort 1 patients remain alive with no evidence of disease clinically or by PET/CT. Of the cohort 2 patients: 1 died 1 had persistent disease 1 remains free of disease. The estimated PFS at 12 months was 88.9% overall, 100% in cohort 1, and 50% in cohort 2. 7/8 patients achieved a complete response (6/7 in cohort 1 and 1/3 in cohort 2), and 1 (cohort 1) achieved partial response (decreased or stable hypermetabolic activity after CRT+MEDI0457) after completion of the immunization series.	All patients cleared detectable HPV DNA at week 16 after immunizations. 5/6 patients cleared HPV RNA by in situ hybridization at the completion of immunization.	8 patients had detectable cellular or humoral immune responses after chemoradiation and MEDI0457. 6 patients showed increased IFN-γ responses over baseline against HPV16 E6 and E7. 5 patients showed increased IFN-γ responses against HPV18 E6 and E7. Anti-HPV responses were numerically greater in cohort 1 (23.3 SFU/10^6^ PBMC to 369 SFU/10^6^ PBMC) compared with cohort 2 (6.7 SFU/10^6^ PBMC to 63.3 SFU/10^6^ PBMC. 6/10 patients exhibited *de novo* sero-responses to HPV16 antigens, and 6/10 patients exhibited *de novo* sero-responses to HPV18 antigens.	Vaccine related AEs in 8 patients–grade 1 injection site bruising (*n* = 2), injection site pain (*n* = 2). Treatment related AEs occurred in 8 patients, mainly grades 1 or 2. Grade >3 AEs in 4 patients–abdominal pain and pneumonia in cohort 1; pathologic fracture, anemia, intestinal perforation (grade 5). were followed after chemoradiation and 3 doses of INO3112.	Expression of PD-L1 on panCK+ tumor cells, CD68+ macrophages, and CD8+ T cells in serial biopsy specimens: post-CRT and post-CRT+MEDI0457 showed decreased epithelial cells, consistent with tumor regression. PD-L1 was detectable on panCK+ tumor cells and CD68+ cells at pre-CRT and post-CRT biopsies. PD-L1 was detectable on CD8+ T cells. Compared with pre-CRT and post-CRT time points, post-CRT þ MEDI0457 biopsies were associated with decreased PD-L1+CD8+, PD-1+CD8+, and PD- L1+CD68+ subpopulations	Too small study (*n* = 10) population, which does not allow for generalization of the results and drawing conclusions. Study included several histologic diagnoses–squamous cell carcinoma, adenocarcinoma, adenosquamous cell carcinoma of the cervix with various prognosis. The confirmation bias-patients received a vaccine 2 to 4 weeks after chemoradiation, which could impact the vaccine effect on organism. It is unclear whether longer period of recovery would result in better outcome. Dosing and timing regimen of MEDI0457 was based on studies of preinvasive cancer, thus the applicability of the regimen for invasive cancer types is questionable [15]. There was no control group of “chemoradiation only” in order to assess the sole effect of vaccination.

Abbreviations:—histopathological—HP; percentage point difference—PPD; progression-free survival—PFS; positron emission tomography—PET; computer tomography—CT; adverse events—AEs; particle-mediated epidermal delivery—PMED; human leukocyte antigens—HLA; chemoradiotherapy—CRT;—intramuscularly—IM; electroporation—EP; cervical biopsy—CB; maximum tolerated dose—MTD; cervical intralesional—IL; calreticulin—C-RT; heat shock proteins—HSPs; squamocolumnar junction—SCJ; viral load—VL.

## Data Availability

The datasets used and/or analyzed during the current study available from the corresponding author on reasonable request.
